# Immune landscape of advanced gastric cancer tumor microenvironment identifies immunotherapeutic relevant gene signature

**DOI:** 10.1186/s12885-021-09065-z

**Published:** 2021-12-11

**Authors:** Simeng Zhang, Mengzhu Lv, Yu Cheng, Shuo Wang, Ce Li, Xiujuan Qu

**Affiliations:** 1 Department of Medical Oncology, the First Hospital of China Medical University, 110001 Shenyang, China; 2 Key Laboratory of Anticancer Drugs and Biotherapy of Liaoning Province, the First Hospital of China Medical University, Shenyang, 110001 China; 3 Liaoning Province Clinical Research Center for Cancer, Shenyang, 110001 China; 4 Key Laboratory of Precision Diagnosis and Treatment of Gastrointestinal Tumors, Ministry of Education, Shenyang, 110001 China; 5 Department of Plastic Surgery, the First Hospital of China Medical University, Shenyang, 110001 China

**Keywords:** Advanced gastric cancer, Tumor microenvironment, Immuno-genomic profiling, WGCNA, ADAMDEC1

## Abstract

**Background:**

Advanced gastric cancer (AGC) is a disease with poor prognosis due to the current lack of effective therapeutic strategies. Immune checkpoint blockade treatments have shown effective responses in patient subgroups but biomarkers remain challenging. Traditional classification of gastric cancer (GC) is based on genomic profiling and molecular features. Therefore, it is critical to identify the immune-related subtypes and predictive markers by immuno-genomic profiling.

**Methods:**

Single-sample gene-set enrichment analysis (ssGSEA) and ESTIMATE algorithm were used to identify the immue-related subtypes of AGC in two independent GEO datasets. Weighted gene co-expression network analysis (WGCNA) and Molecular Complex Detection (MCODE) algorithm were applied to identify hub-network of immune-related subtypes. Hub genes were confirmed by prognostic data of KMplotter and GEO datasets. The value of hub-gene in predicting immunotherapeutic response was analyzed by IMvigor210 datasets. MTT assay, Transwell migration assay and Western blotting were performed to confirm the cellular function of hub gene in vitro.

**Results:**

Three immune-related subtypes (Immunity_H, Immunity_M and Immunity_L) of AGC were identified in two independent GEO datasets. Compared to Immunity_L, the Immuntiy_H subtype showed higher immune cell infiltration and immune activities with favorable prognosis. A weighted gene co-expression network was constructed based on GSE62254 dataset and identified one gene module which was significantly correlated with the Immunity_H subtype. A Hub-network which represented high immune activities was extracted based on topological features and Molecular Complex Detection (MCODE) algorithm. Furthermore, ADAM like decysin 1 (ADAMDEC1) was identified as a seed gene among hub-network genes which is highly associated with favorable prognosis in both GSE62254 and external validation datasets. In addition, high expression of ADAMDEC1 correlated with immunotherapeutic response in IMvigor210 datasets. In vitro, ADAMDEC1 was confirmed as a potential protein in regulating proliferation and migration of gastric cancer cell. Deficiency of ADAMDEC1 of gastric cancer cell also associated with high expression of PD-L1 and Jurkat T cell apoptosis.

**Conclusions:**

We identified immune-related subtypes and key tumor microenvironment marker in AGC which might facilitate the development of novel immune therapeutic targets.

**Supplementary Information:**

The online version contains supplementary material available at 10.1186/s12885-021-09065-z.

## Background

Gastric cancer (GC) is the third leading cause of cancer death and the fifth most common malignancy worldwide [1]. Due to the lack of effective screening to detect early-stage GC, most patients are diagnosed with AGC which has poor prognosis [2]. Although improved therapeutic strategies have been developed, clinical outcomes remain unsatisfactory. Recently, immune checkpoint blockades such as anti-programmed cell-death protein 1(PD1) or programmed cell-death 1 ligand 1 (PD-L1) drugs have been widely used and achieved efficacy in different cancer types [3, 4]. Anti-PD-1/PD-L1 therapy has also been shown to be effective and is approved for third-line treatment in metastatic GC [5]. However, only some subsets of GC patients benefit from immunotherapy. PD-L1 expression, Epstein–Barr infection and microsatellite status have been reported to be associated with immunotherapeutic responsiveness [6, 7]. However, the dominant population and prognostic marker of immunotherapies are currently unknown.

The tumor immune microenvironment (TME) consisting of immune and stromal cells has been proven to be associated with clinical outcomes to immunotherapy [8, 9]. In GC, the immune microenvironment has a complex relationship with cancer occurrence and progression, which could be regulated by Tumor-infiltrating immune cells (TIICs) [10, 11]. An increasing number of studies have indicated that TIICs play important roles as prognostic markers and are potential therapeutic targets [12, 13]. The Estimation of Stromal and Immune Cells in Malignant Tumors using Expression data (ESTIMATE) algorithm which is based on single sample gene set enrichment analysis, evaluated the immune and stromal infiltration level in tumors by calculating immune, tumor purity and stromal scores [14]. Recently, the ESTIMATE algorithm has been wildly used to investigate the TME in acute myeloid leukemia, colorectal cancer and breast cancer [15–17]. However, the roles of TIICs in AGC and hub-genes correlations with the TME remain to be fully understood.

In this study, AGC was classified into three distinct subtypes by immuno-genomic profiling and confirmed the reliability of classification model in two independent Gene Expression Omnibus (GEO) datasets. We further identified the hub genes and critical pathways in different immune subtypes of AGC. The results may offer novel evidence in predicting prognosis and immunotherapeutic targets of AGC.

## Methods

### Data collection

Microarray data of GSE62254 and GSE29272 were obtained from the Gene Expression Omnibus (www.ncbi.nlm.nih.gov/geo/). The data of GSE62254 was based on GPL570 platforms (Affymetrix Human Genome U133 Plus 2.0 Array, 300 GC patients). The GSE29272 data was based on GPL96 platforms which included 268 GC patients. Two hundred ninety-five samples of GSE62254 and 126 samples of GSE29272 with both gene expression data and clinical parameters of advanced gastric cancer were included. Kaplan Meier-plotter (KMplotter) (http://www.kmplot.com/) was used for external validation.

### Data clustering and evaluation of immune and stromal scores

Single-sample gene-set enrichment analysis (ssGSEA) was used to evaluate the enrichment levels of the 29 immune signatures and hierarchical clustering was performed according to the ssGSEA score [18, 19]. ESTIMATE algorithm which integrated in “estimate” R package in R version 3.6.2. was applied to measure immune microenvironment infiltration based on gene expression data [14].

### Proportions of immune cell subsets between GC immune subtypes

CIBERSORT [20] was used to estimate the proportions of 22 immune cell subsets and the relative expression of 22 immune cell subsets in each sample was determined. *P* < 0.05 was set as criteria for subsequent analysis.

### Gene-set enrichment analysis

Gene-set enrichment analysis of each GEO datasets was applied to identify the Gene Ontology (GO) terms and Kyoto Encyclopedia of Genes and Genomes (KEGG) pathways participating in high immunity subgroup of advanced gastric cancer. FDR < 0.05 was set as criteria to select significance pathways.

### Identification of immune subtype-specific genes in advanced gastric cancer

Weighted Gene Co-expression Network Analysis (WGCNA) was used to construct gene co-expression network and extract the gene information in each module [21]. The correlation between module eigengenes and immune subtypes was evaluated by Pearson’s correlation tests. To explore the potential biological process of genes within the immune-related modules, GO and KEGG enrichment analysis were performed and visualized by clusterprofiler package in R project [22].

### Protein-protein interaction network construction

Protein-protein interaction network (PPI) was constructed by Cytoscape software (v3.6.1) [23]. The hub network was selected by topological features. MCODE (Molecular Complex Detection) algorithm was used to further identify the hub genes in the PPI network [24].

### Genomic and clinical data with immunotherapy

The value of ADAMDEC1 in predicting immunotherapeutic response was analyzed by IMvigor210 datasets. The expression profile and clinical parameter of IMvigor210 dataset that is available under the Creative Commons 3.0 license was downloaded from http://research-pub.gene.com/IMvigor210CoreBiologies. A total of 298 urothelial cancer samples with both gene expression and immune response parameter were selected to further analysis.

### Survival analysis

Survival curves were plotted by the Kaplan-Meier (KM) method and compared with the log-rank test. The expression level of hub-genes was separated to high and low expression according to the median value. *P* < 0.05 considered as a threshold to identify the significance genes associated with prognosis of advanced gastric cancer.

### Experimental validation

#### Cell culture

MGC803 gastric cancer cell line was obtained from the Type Culture Collection of the Chinese Academy of Sciences (Shanghai, China). Jurkat T cells were obtained from the American Type Culture Collection (ATCC, Rockville, MD, USA). All cells were grown in RPMI-1640 (GibcoBRL, USA) supplemented with 10% fetal bovine serum (FBS), penicillin (100 U/mL) and streptomycin (100 mg/ mL), in a humid atmosphere containing 5% CO 2 at 37 °C.

#### Reagents and antibodies

Anti-ADAMDEC1 antibodies were obtain form Novus Biologicals (USA). Antibodies specific to PD-L1 (13684S) was from Cell Signaling Technology (Danvers, MA, USA). All the other antibodies were purchased from Santa Cruz Biotechnology (USA).

#### Small interfering RNA (siRNA) transfections

The ADAMDEC1 siRNA sequences from Beijing GeneX Health technology Co., Ltd. (Beijing, China), were used: 5′-GCCTGTACTTTGGCTCATTGTTCTT-3′. The siRNAs were transfected with Lipofectamine 2000 (Invitrogen, Carlsbad, CA) per the manufacturer’s instructions.

#### MTT assay, Transwell migration assay and Western blotting

MTT assay, migration assay and Western blot and were performed as described previously [25].

### Real-time PCR analysis

The real-time PCR conditions included initial activation at 95 °C for 5 min, followed by 15 s of 45 cycles at 95 °C and 60 °C for 1 min (Applied Biosystems® 7500 Real-Time PCR Systems, Thermo fisher, IL, USA). Primer sequences for PD-L1: Forward (5′-TTTCAATGTGACCAGCAC-3′), Reverse (5′- GGCATAATAAGATGGCTC-3′); 18S: Forward (5′-CCC GGG GAG GTAGTG ACG AAA AAT-3′), Reverse (5′-CGCCCGCCCGCTCCCAAGAT-3′).

### Cell apoptosis assay

Jurkat T cells (3 × 10^5^/well) were co-cultured with MGC803 cells for 48 h. After that, Jurkat T cells were harvested and stained using an Annexin V-fluorescein isothiocyanate/propidium iodide apoptosis detection kit (BMS500FI-100; Invitrogen; Thermo Fisher Scientific, Inc.) and the number of apoptotic T cells was determined by FACSCalibur flow cytometry (BD Biosciences, San Jose, CA, USA) according to the protocol. The samples were selected and analyzed by BD Accuri C6.

#### Statistical analysis

Data are reported as means ± SD. Student’s t-test or one-way ANOVA were applied to evaluate differences between or among groups. *P* < 0.05 was determined statistically significant. Each experiment was repeated at least three times.

## Results

### Identification of immune subtypes in AGC by immuno-genomic profiling

**Fig. 1:**
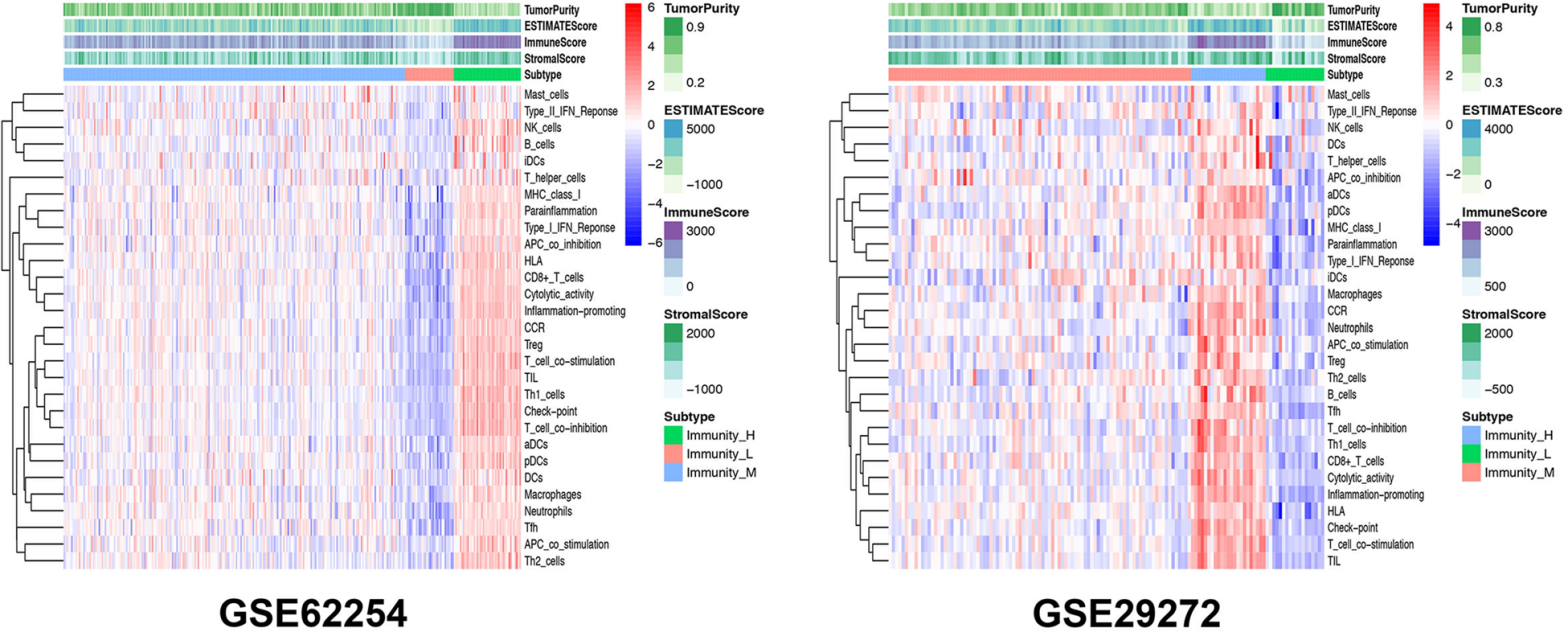
Clustering of immune-related subtypes of AGC. Three immune-related subtypes of advanced gastric cancer in two independent datasets were generated by Hierarchical clustering. Immunity_L, Immunity_M and Immunity_H refers to Immuunity Low, Immunity Medium and Immunity High respectively. ImmuneScore, StromalScore and TumorPurity were calculated by ESTIMATE algorithm

**Fig. 2:**
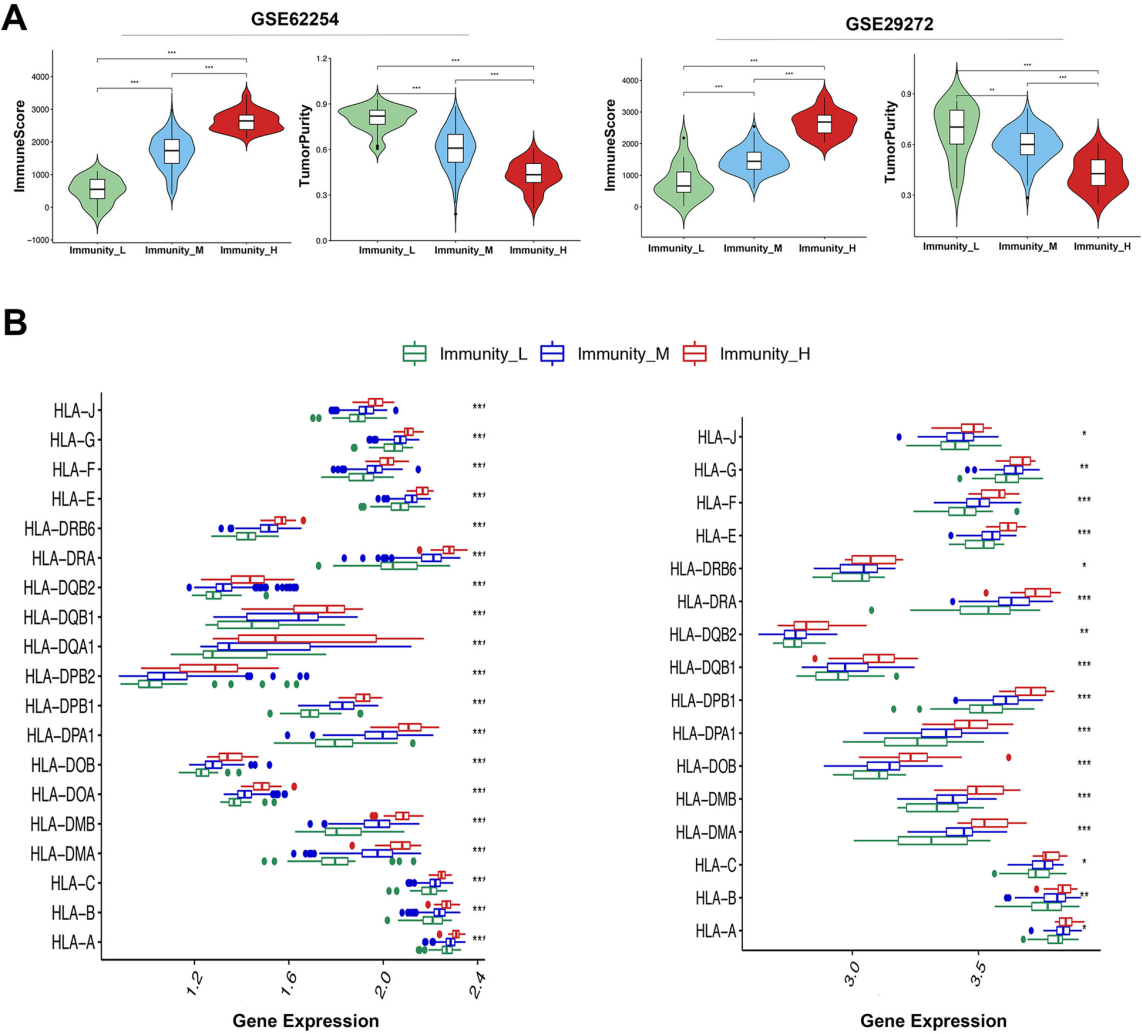
Characteristics of different immune-related subtypes. **A** The level of immune cell infiltration in different immune subtypes (MannWhitney U test). **B** Comparison of HLA genes expression between different immune subtypes (ANOVA test)

AGC data were obtained from GSE62254 and GSE29272 datasets. Samples with both gene expression data and clinical parameters were selected for further analysis (Table 1). To quantify the enrichment of immune cells and pathways in each GC sample, we analyzed 29 immune-related gene sets (Table S1) and calculated ssGSEA scores. The results of hierarchical cluster showed three clusters that were separated in the GSE62254 and GSE29272 datasets (Fig. 1). According to the ssGSEA score, clusters were defined as Immunity Low (Immunity_L), Immunity Medium (Immunity_M) and immunity High (Immunity_H) (Table 2). Using the ESTIMATE algorithm, we calculated immune scores and tumor purity for all samples. Compared to Immunity_L subtype, the immune scores were higher in the Immunity_H (Fig. 2A) subgroup and similar trends were obtained in stromal score in GSE62254 (Additional file 1: Fig. S1A). In contrast, tumor purity scores were lower in the Immunity_H subgroup. These results indicated that immune cells were highly infiltrative in the Immunity_H subgroup and tumor cells were more detected in the Immunity_L subgroup.

**Table 1:** Characteristics of GSE62254 and GSE29272 cohort

GSE62254		GSE29272		
Characteristic	Number of Patients (%)	Characteristic	Number of Patients (%)	
**Age (years)**		**Age (years)**		
Median (Range)	63 (24–86)	Median (Range)	59 (23–71)	
**Gender**		**Gender**		
Male	195 (66.1%)	Male	99 (78.6%)	
Female	100 (33.9%)	Female	27 (21.4%)	
**T stage**		**TNMstage**		
T2	184 (62.4%)	I	5 (4.0%)	
T3	90 (30.5%)	II	5 (4.0%)	
T4	21 (7.1%)	III	108 (85.7%)	
**N stage**		IV	8 (6.3%)	
N0	38 (12.9%)			
N1	128 (43.4%)			
N2	79 (26.8%)			
N3	50 (16.9%)			
**M stage**				
M0	268 (90.8%)			
M1	27 (9.2%)			
**TNMstage**				
I	30 (10.2%)			
II	94 (31.9%)			
III	95 (32.2%)			
IV	76 (25.8%)			
**Lauren**				
Intestinal	144 (48.8%)			
Diffuse	134 (45.4%)			
Mixed	17 (5.8%)

**Table 2:** Clinical Characteristics of Immune Subtypes

GSE62254							GSE29272						
	Immunity L		Immunity M		Immunity H			Immunity L		Immunity M		Immunity H	
	No.	%	No.	%	No.	%		No.	%	No.	%	No.	%
**Age**							**Age**						
< 60	10	9.4%	79	74.5%	17	16.0%	< 60	11	16.4%	41	61.2%	15	22.4%
≥60	22	11.6%	140	74.1%	27	14.3%	≥60	7	11.9%	48	81.4%	4	6.8%
**Gender**							**Gender**						
Male	24	12.3%	143	73.3%	28	14.4%	Male	14	14.1%	70	70.7%	15	15.2%
Female	8	8.0%	76	76.0%	16	16.0%	Female	4	14.8%	19	70.4%	4	14.8%
**pStage**							**pStage**						
I/II	14	11.3%	93	75.0%	17	13.7%	I/II	2	20.0%	8	80.0%	0	0.0%
III/IV	18	10.5%	126	73.7%	27	15.8%	III/IV	16	13.8%	81	69.8%	19	16.4%
**Metastasis**													
Yes	16	11.3%	110	77.5%	16	11.3%							
No	16	10.5%	109	71.2%	28	18.3%

Association between the HLA genes and immune subtypes was further investigated. Interestingly, the expression level of HLA genes was notably higher in the Immunity_H compared to the Immunity_L (ANOVA test, *P* < 0.05) (Fig. 2B). Furthermore, we evaluated proportions of the 22 TIICs in each sample using CIBERSORT plug-in in R package [20]. The fractions of M1 Macrophages, activated memory CD4 T cells, CD8 T cells and γδT cells were significantly higher in the Immunity_H subtype in both GEO datasets as shown in Fig. S1B.

**Fig. 3:**
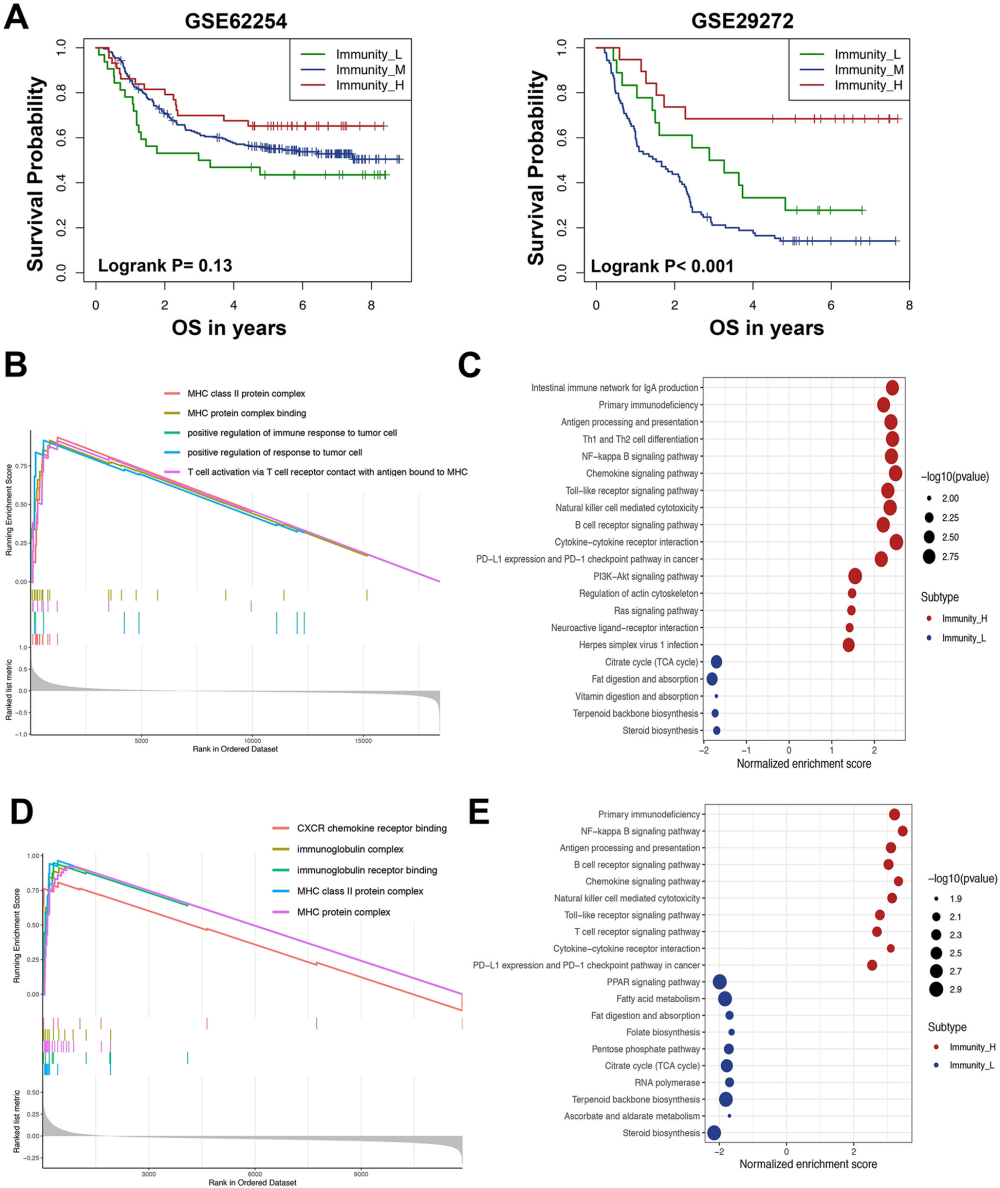
Gene set enrichment analysis of immune-related subtypes. **A** Overall survival of different immune subtypes by Kaplan-Meier analysis (log-rank test *P* = 0.13 in GSE62254; *P* < 0.001 in GSE29272). **B** GSEA GO analysis of Immunity_H subtypes in GSE62254. **C** KEGG enrichment analysis of Immunity_H and Immunity_L subtypes in GSE62254. **D** GSEA GO analysis of Immunity_H subtypes in GSE29272. **E** KEGG enrichment analysis of Immunity_H and Immunity_L subtypes in GSE29272

The prognostic values of immune subtype were analyzed by survival analysis. Compared to the Immunity_L, the Immunity_H subtype had a significantly higher survival in GSE29272 (log-rank *P* < 0.001). Although there has no statistical significance in GSE62254 (log-rank *P* = 0.13), the Immunity_H still showed improved survival outcome compared to the Immunity_M and Immunity_L subtypes (Fig. 3A). To further confirm the prognostic value of Immuntiy_H subtype, we analyzed the prognostic difference between Immunity_H and Immunity_L subtypes in two independent datasets. As show in Fig. S1C, the Immunity_H presents significantly better OS compare to Immunity_L both in GSE62254 (log-rank *P* = 0.047) and GSE29272(log-rank *P* < 0.001). These findings showed that high immune activity could be associated with better clinical outcomes in AGC.

### Gene Set Enrichment Analysis (GSEA) of immune subtypes

To explore the potential mechanism participating in high immune activity subtype in AGC, a number of GO terms and KEGG pathways were identified in Immuntiy_H by GSEA. In GSE62254, the MHC class II protein complex, the MHC protein complex binding, positive regulation of immune response to tumor cells and positive regulation of response to tumor cells were the most significantly enriched cellular functions (Fig. 3B). CXCR chemokine receptor binding, the immunoglobulin complex, the immunoglobulin receptor binding, the MHC class II protein complex and the MHC protein complex were significantly enriched in GSE29272 (Fig. 3D). KEGG pathway enrichment analysis indicated that immune-associated pathways were highly enriched in the Immunity_H subgroup in both GSE62254 and GSE29272, which included primary immunodeficiency, antigen processing and presentation, the B cell receptor signaling pathway, chemokine signaling, natural killer cell-mediated cytotoxicity, the Toll-like receptor signaling pathway, PD-L1 expression and the PD-1 checkpoint pathway in cancer (Fig. 3C, E). These results confirmed that immune activity was up-regulate in the Immuntiy_H subtype. In contrast, citrate cycle, fat digestion and absorption and steroid biosynthesis were enriched in the Immunity_L subgroup suggesting that these pathways could be negatively correlated with immune activity in AGC.

### Association between immune subtypes and traditional classification

**Fig. 4:**
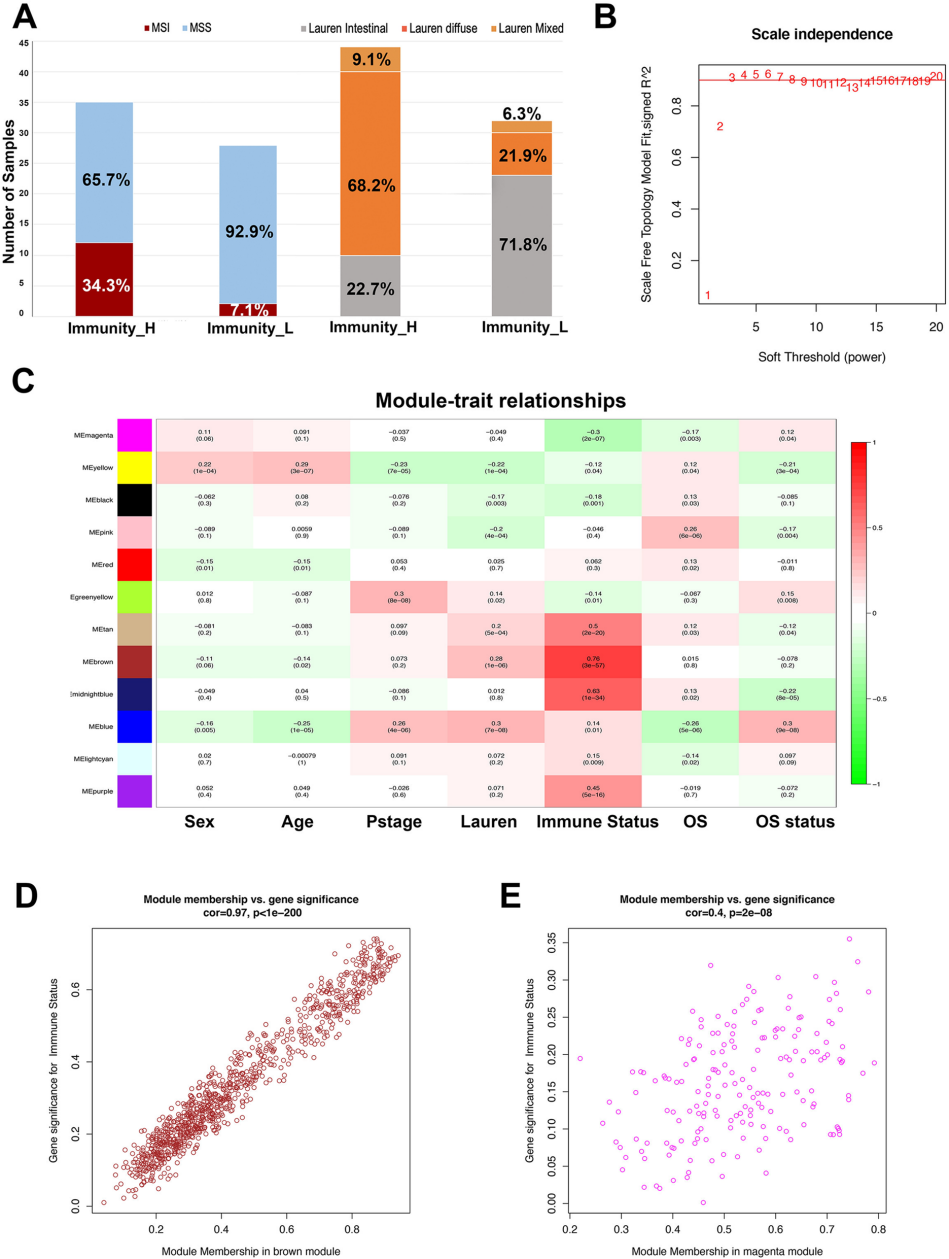
Construction of co-expression network of immune-related subtypes. **A** Comparison of the immune-related classification and traditional classification of gastric cancer in GSE62254 and Immunity_H was highly correlated with MSI (Fisher’s exact test, *p* = 0.022) and lauren diffuse subtype (Fisher’s exact test, *p* < 0.001). **B** The correlation of different soft threshold power values of co-expression network. **C** Module-trait associations were evaluated by correlations between module eigengene and clinical traits. **D** The correlation of genes in brown module with immune subtypes trait. **E** The correlation of genes in magenta module with immune subtypes trait

We compared the immune subtypes and traditional classification of GC in GSE62254. The results showed that Immunity_H was highly correlated with MSI (Fisher’s exact test, *p* = 0.022) and Lauren diffuse subtype (Fisher’s exact test, *p* < 0.001) (Fig. 4A). In the Immunity_L subgroup, MSS and the Lauren intestinal subtypes were mostly detected. Recent studies have indicated that MSI was associated with immunotherapeutic response in GC [6]. In addition, non-intestinal histology by Lauren classification was associated with higher degree of host immune response [26].

### Identification of vital candidate markers of immune subtypes

**Fig. 5:**
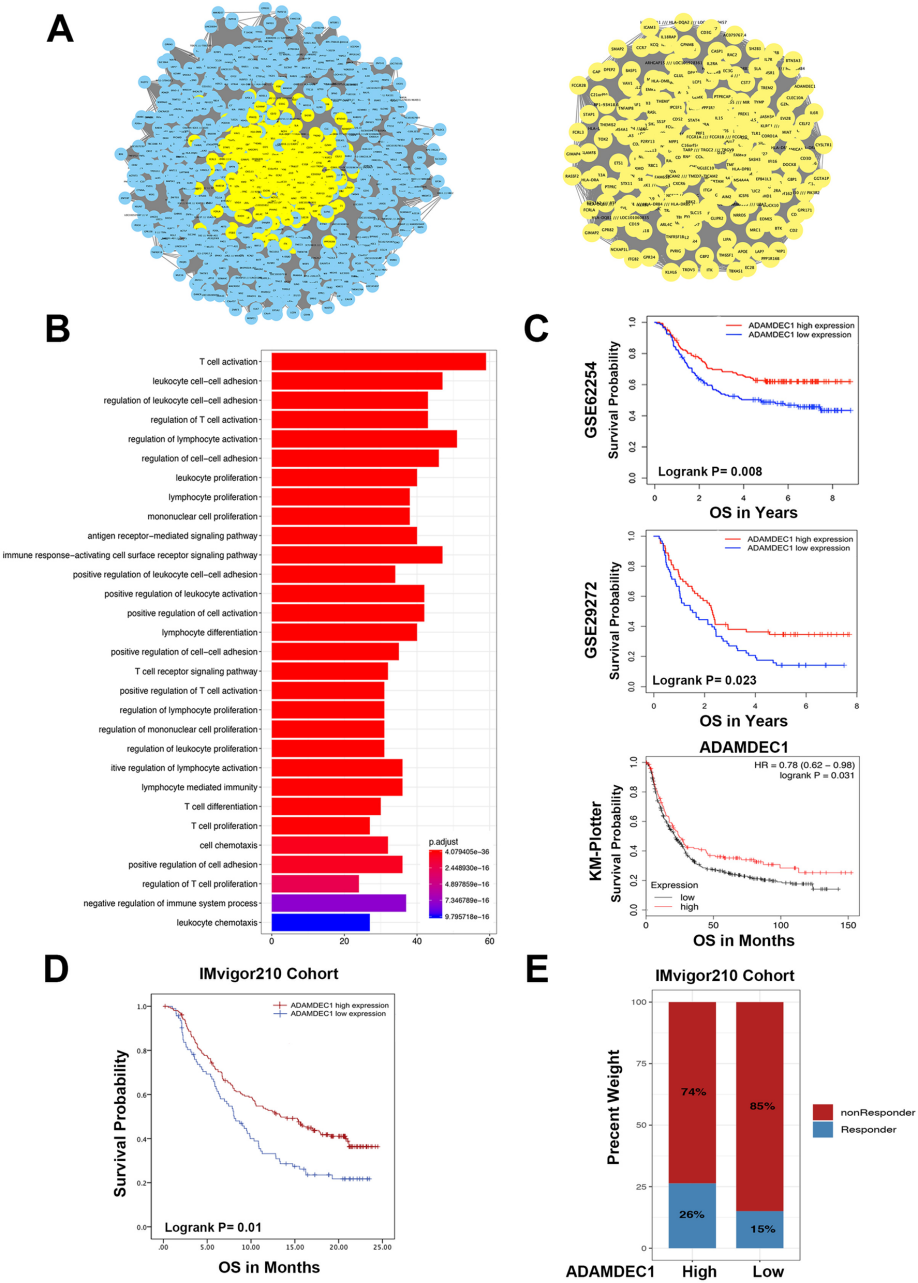
Identification of the hub-network and hub-genes in Immunity_H subtype. **A** PPI network of genes in brown module which including 955 nodes and 248,141 edges. Hub-network was extracted from PPI network according to topological features and MCODE algorithm which consist of 275 nodes and 37,675 edges. **B** Significantly enriched KEGG pathways of hub-network genes. **C** Overall Survival (OS) of ADAMDEC1 in GSE62254 cohort by Kaplan-Meier (KM) analysis (log-rank *P* < 0.008) and external validation by GSE29272 and KMplotter cohort (log-rank *P* = 0.023, *P* = 0.031, respectively). **D** Overall survival of high and low ADAMDEC1 expression by Kaplan-Meier analysis in IMvigor210 cohort (Log rank test, *p* = 0.01). **E** Rate of clinical response to anti-PD-L1 immunotherapy in high and low ADAMDEC1 expression groups in IMvigor210 cohort

To further investigate candidate markers associated with immune activity in AGC, we constructed a gene co-expression network of the GSE62254 dataset by WCGNA. In the process of constructing the network, the power value is a vital parameter that can affect the independence and average connectivity degree of the co-expression modules. In order to make the co-expression network approximate to scale-free topology distribution, the soft-thresholding power equals 3 was selected (Fig. 4B, Fig. S1D). Among of 16 modules, four pairs of gene modules were merged with high adjacency degree base on the threshold 0.2(Fig. S1E) and a total of 12 modules were identified to further analysis. Correlation between immune subtypes and module eigengene was analyzed, which shows the brown, midnight blue, tan and purple modules were positively associated with immune status. In contrast, the black, magenta, yellow and greenyellow modules were negatively associated with immune traits (Fig. 4C). To identify the critical genes for immune subtypes in AGC, the brown module that was the most strongly correlated module to the Immunity_H was selected (Fig. 4D, E). A protein-protein network (PPI) of the brown module was constructed consisting of 955 nodes and 248,141 edges. Parameters of degree, betweenness and closeness were used to describe the topological features of the PPI network. Genes with degree > 750 (2 folds of median value 375), betweenness > 5.7e-6 (Median value) and closeness > 0.622 (Median value) were selected to construct the hub-network including 275 nodes and 37,675 edges (Fig. 5A) (Table S2). The Molecular Complex Detection (MCODE) was applied to screen the hub-cluster. Interestingly, the top significant cluster was consistent with the hub-network, indicating that these 275 genes were highly correlated with the Immunity_H subtype. Furthermore, ssGSEA score was recalculated base on 275-gene signature and the cluster heatmap showed high expression of 275 hub-genes are strongly correlated with Imunity_H subtype and high immune cell infiltration (Fig. S2). The enrichment analysis of biological processes of 275 genes showed that immune-associated processes were significantly enriched (Fig. 5B). Among hub-gene network, the gene which have the highest Neighborhood Connectivity, Degree and MCODE score was confirmed to be the seed gene. ADAMDEC1 was selected to further analysis as the seed gene in 275 hub-genes (Table S3). Kaplan-Meier analysis of overall survival (OS) demonstrated that high levels of ADAMDEC1 were significantly associated with better prognosis in both GSE62254 (log-rank *P* = 0.008) and external validation of GSE29272 (log-rank *P* = 0.023) and KMplotter (log-rank *P* = 0.031) (Fig. 5C).

### The role of ADAMDEC1 in the prediction of immunotherapeutic response

In order to investigate the value of ADAMDEC1 in speculating the therapeutic response, the samples who received immunotherapy in the IMvigor210 cohort were selected to further analysis. In a total of 298 urothelial cancer samples, high level of ADAMDEC1 expression showed significant better OS compared to low expression group (log-rank *P* = 0.01). We also found that high expression of ADAMDEC1 was correlated with objective response to anti-PD-L1 therapy (Fisher’s exact test, *p* = 0.031) (Fig. 5D-E). These findings suggested the predictive value of ADAMDEC1.

### Experimental validation by gastric cancer cells

**Fig. 6:**
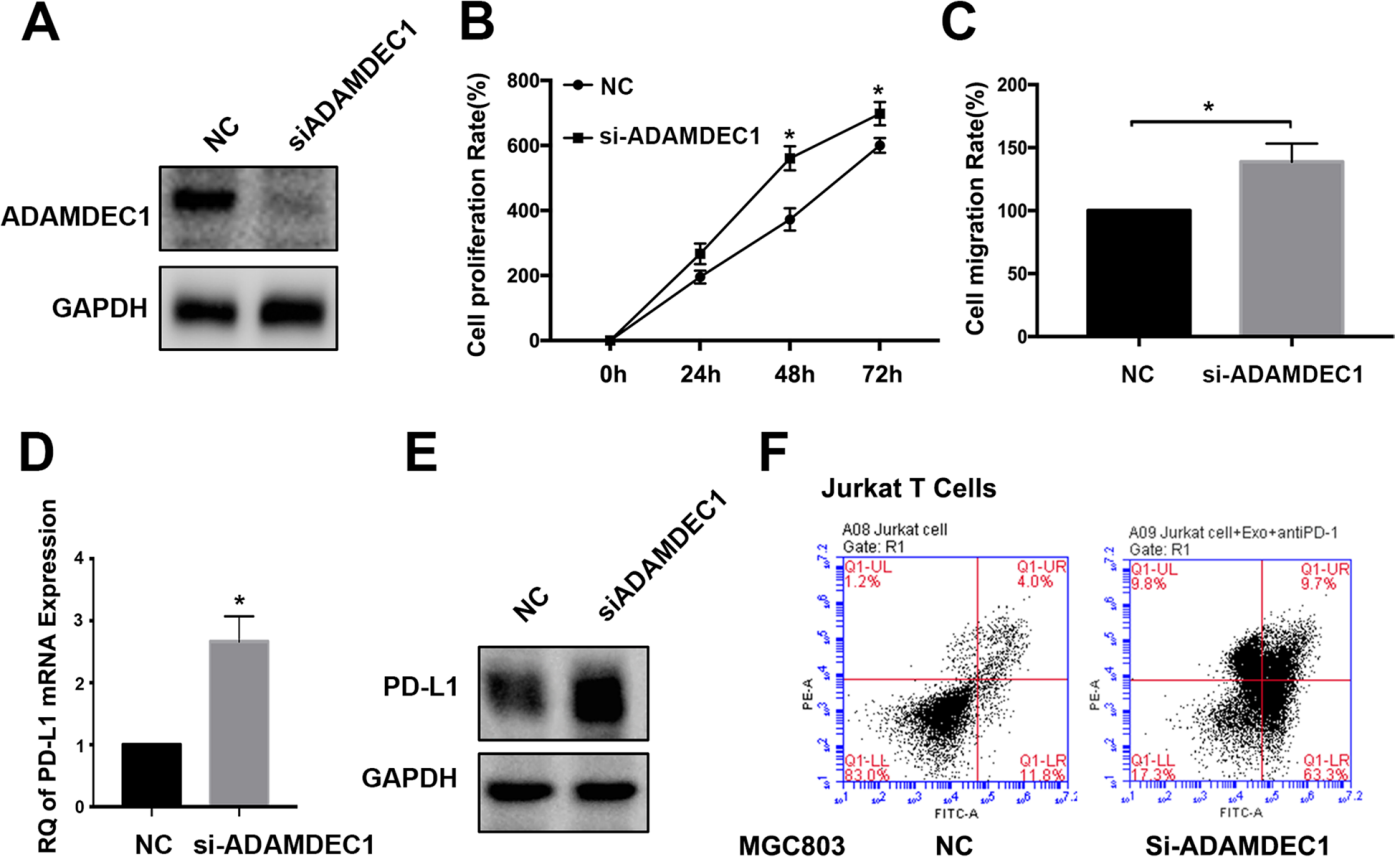
Experimental validation by gastric cancer cells. **A** MGC803 cell was knockdown of ADAMDEC1 gene and western blot was applied to detect the expression level of ADAMDEC1. **B** MTT assay was used to detect the cell proliferation rates in 0 h, 24 h, 48 h and 72 h. Data are means ± SD in three independent experiment (*P < 0.05). **C** Transwell assay was performed to detect the migration of MGC803 cell after silencing ADAMDEC1 for 48 h. Data are means ± SD in three independent experiment (*P < 0.05). **D** mRNA expression level change of PD-L1 in silencing ADAMDEC1 of MGC803 cell. **E** Western blot was used to detect the change of PD-L1 expression level in MGC803 cell with ADAMDEC1 knockdown. **F** Jurkat T cells were co-incubated with ADAMDEC1-NC and ADAMDEC1-KD MGC803 cell for 48 h, respectively. The apoptosis in Jurkat T cells was measured by flow cytometry analysis

To further confirm the potential function of ADAMDEC1 in MGC803 gastric cancer cell line, silencing of ADAMDEC1 expression by siRNA was conducted (Fig. 6A). The results demonstrated that deficiency of ADAMDEC1 promoted gastric cancer cell proliferation and migration as presented in Fig. 6B, C. Interestingly, PD-L1 mRNA and expression levels were both upregulated following with depletion of ADAMDEC1 in MGC803 cell line (Fig. 6D, E). Besides, apoptosis in Jurkat T cells was enhanced significantly after co-incubated with ADAMDEC1 silencing MGC803 cell (Fig. 6F). These findings suggested that ADAMDEC1 was a critical marker in predicting proliferation and immune response in gastric cancer cells.

## Discussion

AGC remains a major clinical problem with poor prognosis due to the limited effectiveness of therapies. Although immune checkpoint blockades provide a treatment paradigm, only a small number of patients may benefit from treatment. Current classifications of GC are mostly based on genomic analysis or molecular features. Recently, studies have focused on GC classification based on immune profiling [27–29]. In the present study, GC was classified into three immune-related subtypes, which included Immunity_H, Immunity_M and Immunity_L. The immunity high subtype was positively correlated with immune score and negatively correlated with tumor purity, which showed that Immunity_H cancers were strongly infiltrated by immune cells and had high immune activities. Immunity_H also indicated higher immunogenicity compared to the other subtypes because of high expression of HLA genes. In addition, the proportions of 22 immune gene signatures were calculated by CIBRTSORT. Macrophages M1, CD4 T cells, CD8 T cells and γδT cells were both highly presented in the Immunity_H subtype of the two independent datasets which further suggested increased anti-tumor immune activity in the immunity_H subgroup. Survival analysis confirmed favorable prognosis of the Immunity_H subtype in GSE29272. Whilst there was no significant difference in overall survival between the subtypes in GSE62254, the survival curve showed the same trend in GSE29272. These results are consistent with numerous previous studies which have showed better prognosis in the high immune cell infiltration group [13, 30].

Gene ontology analysis showed that the MHC terms were enriched in Immunity_H subtype of both GSE datasets. Furthermore, we found that the immunity high subtype was highly enriched in immune signatures such as primary immunodeficiency, antigen processing and presentation, B cell receptor signaling pathway, PD-L1 expression and PD-1 checkpoint pathway in cancer. In addition, cancer-associated pathways including PI3K-AKT and RAS signaling were also enriched in the immunity high subtype. Previous studies showed that PI3K-AKT and RAS signaling pathways have participated in multiple immunity processes in the tumor [31–33], suggesting the potential roles of these pathways in regulating immune activity in AGC. In contrast, immune-related pathways were not frequently enriched in the Immunity_L subtype.

Comparison of immune subtypes and traditional classification in GC indicated that the Immunity_H subtype was associated with MSI and the lauren diffuse type. MSI has been confirmed to be part of the sensitive index to anti-PD-1/PD-L1 treatment. Thus, these findings suggest that the Immunity_H subtype of AGC might benefit from immunotherapy.

To further identify the critical markers of the Immunity_H subtype, we constructed a co-expression network of the GSE62254 dataset by WGCNA. The Brown module was mostly correlated with the Immunity_H subtype. The hub-network was constructed by calculating the topological features of the PPI network of the brown module. GO analysis of the hub-network genes indicated that immune-associated biological processes were highly enriched which are represented by T cell activation and immune response. Based on the MCODE algorithm, 275 genes within the hub-network contributed equally to the network and accurately represented the Immunity_H subtype. Among the 275 genes, ADAMDEC1 was marked as seed gene.

ADAM like decysin 1 (ADAMDEC1) is a member of the ADAM family of metalloproteinases. Unlike the other ADAM family member, ADAMDEC1 lack of a transmembrane domain and altered catalytic domain [34, 35]. In the current study, ADAMDEC1 was confirmed to be associated with favorable prognosis in gastric cancer. Several studies have shown that ADAMDEC1 as a prognostic factor in gastric adenocarcinoma and the mRNA expression of ADAMDEC1 is decreased during both tumorigenesis and tumor progression in colorectal cancer [36, 37]. In vitro, ADAMDEC1 could negatively regulate GC cells proliferation and migration. In addition, we found that depletion of ADAMDEC1 increased PD-L1 expression in gastric cancer cell. A systematic review and meta-analysis confirmed that PD-L1 overexpression is a significant adverse prognostic factor in gastric cancer [38]. Besides, ADAMDEC1 has been reported to function in regulating the immune response and might play an important role in dendritic cell function [35]. The patients who receiving immunotherapy were evaluated by IMvigor210 datasets as the independent validation [39], we notice that the expression level of ADAMDEC1 was significantly upregulated in patients responding to immunotherapy and survival benefit also detected in high ADAMDEC1 group. Furthermore, silencing ADAMDEC1 of gastric cancer cells promoted apoptosis of Jurkat T cells. Collectively, ADAMDEC might serve as biomarker of prognosis and immune response in AGC.

## Conclusions

In summary, classification based on immune signatures reflected immune activity in different subtypes in AGC. ADAMDEC1 served as hub-gene was identified and validated to confirm predictive values in immune activity and prognostic values in AGC patients. The mechanism regulating the TME and clinical application value of ADAMDEC1 require further investigation. This study can potentially provide novel biomarker and therapeutic targets in AGC.

## Supplementary Information

**Additional file 1: Supplementary Figure 1.** (A) The level of Stromal score in different immune subtypes (MannWhitney U test). (B) Comparison of the fraction of immune cell subtypes between different immune subtypes (Kruskal-Wallis test). *P < 0.05; **P < 0.01; ***P < 0.001. (C) Overall survival of Immunity_H and Immunity_L subtypes by Kaplan-Meier analysis (log-rank test *P* = 0.047 in GSE62254; *P* < 0.001 in GSE29272). (D) Identification of the best soft-thresholding power value. (E) 4 pairs of gene modules were merged due to their similarity according to the threshold (Red line). (F) The gene dendrogram was constructed by hierarchical clustering base on dissTOM of genes. (G) Heatmap plot of the adjacencies of modules. **Supplementary Figure 2.** (A, B) High expression level of 275 hub-genes was correlated with Immunity_H subtype and high immune cell infiltration. ***P < 0.001.

**Additional file 2.**

**Additional file 3.**

**Additional file 4.**

## Data Availability

The datasets used and/or analysed during the current study are available in TCGA (https://portal.gdc.cancer.gov) and GEO datasets (GSE62254: https://www.ncbi.nlm.nih.gov/geo/query/acc.cgi?acc=GSE62254; GSE29272: https://www.ncbi.nlm.nih.gov/geo/query/acc.cgi?acc=GSE29272) and IMvigor210 datasets: http://research-pub.gene.com/IMvigor210CoreBiologies.

## Abbreviations

AGC: Advanced gastric cancer
ssGSEA: Single-sample gene-set enrichment analysis
WGCNA: Weighted gene co-expression network analysis
MCODE: Molecular Complex Detection
TME: Tumor immune microenvironment
ESTIMATE: Estimation of Stromal and Immune Cells in Malignant Tumors using Expression data
GEO: Gene Expression Omnibus
GO: Gene Ontology
KEGG: Kyoto Encyclopedia of Genes and Genomes
ADAMDEC1: ADAM like decysin 1
