# Supplementary figures and images for: Immune landscape of advanced gastric cancer tumor microenvironment identifies immunotherapeutic relevant gene signature
Source: BMC Cancer. 2021 Dec 11;21:1324. doi: 10.1186/s12885-021-09065-z (PMC8665569; doi:10.1186/s12885-021-09065-z)

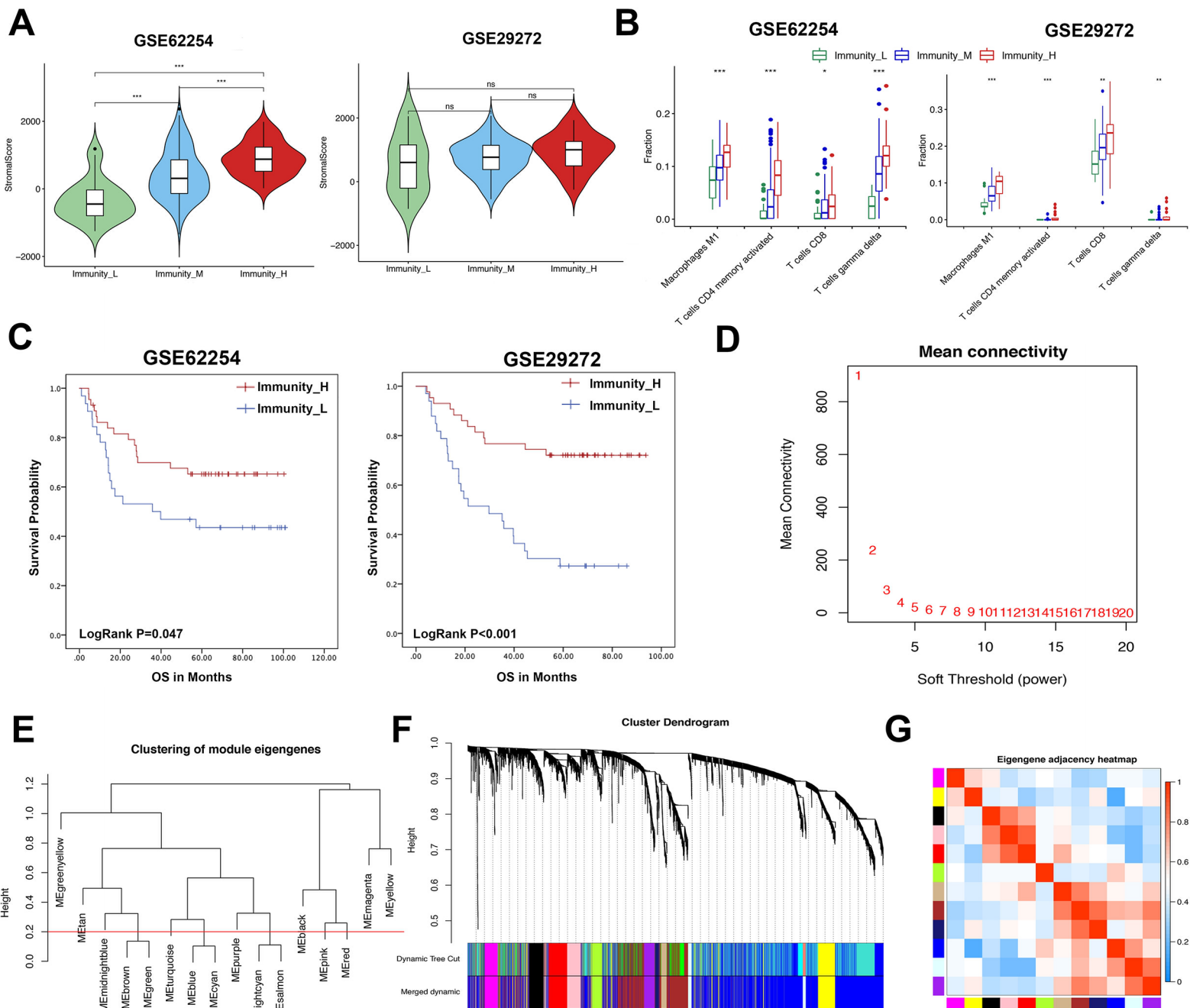

A

GSE62254

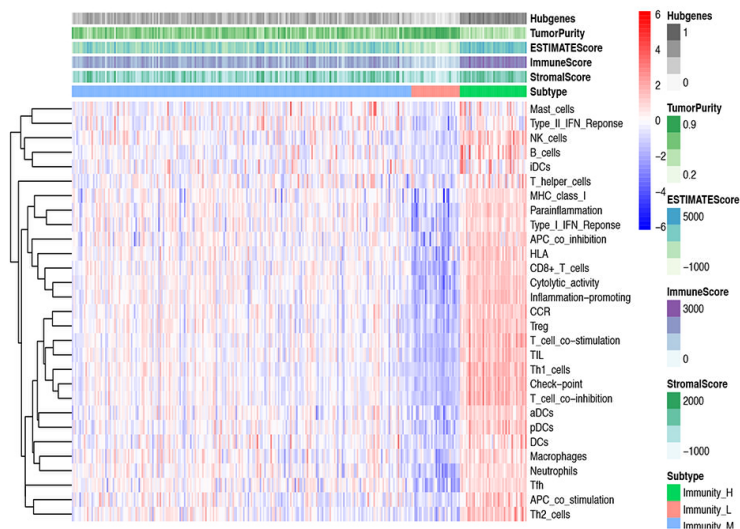

GSE29272

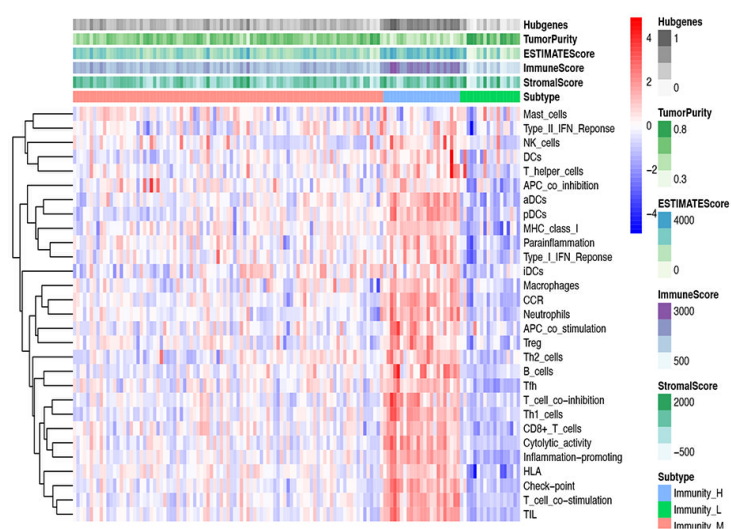

B

GSE62254

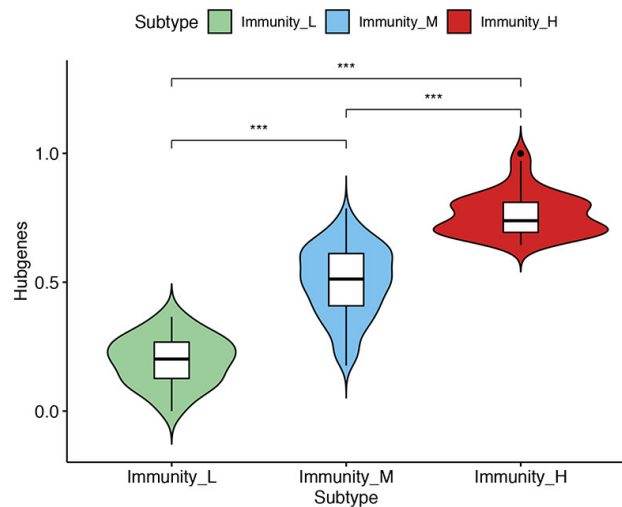

GSE29272

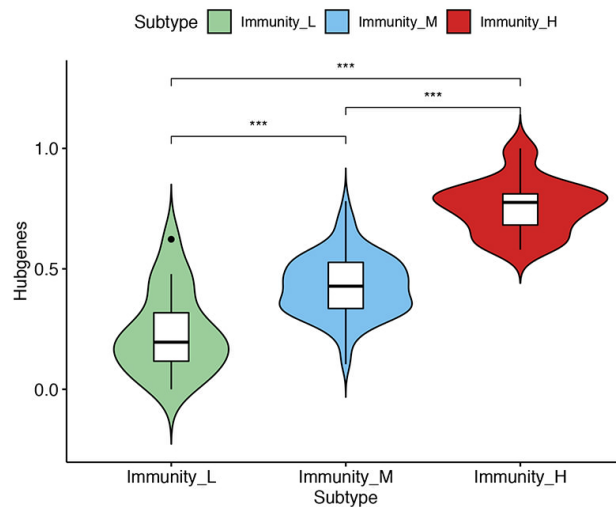

Supplement: Supplementary file 1 — Additional file 1: Supplementary Figure 1. (A) The level of Stromal score in different immune subtypes (MannWhitney U test). (B) Comparison of the fraction of immune cell subtypes between different immune subtypes (Kruskal-Wallis test). *P < 0.05; **P < 0.01; ***P < 0.001. (C) Overall survival of Immunity_H and Immunity_L subtypes by Kaplan-Meier analysis (log-rank test P = 0.047 in GSE62254; P < 0.001 in GSE29272). (D) Identification of the best soft-thresholding power value. (E) 4 pairs of gene modules were merged due to their similarity according to the threshold (Red line). (F) The gene dendrogram was constructed by hierarchical clustering base on dissTOM of genes. (G) Heatmap plot of the adjacencies of modules. Supplementary Figure 2. (A, B) High expression level of 275 hub-genes was correlated with Immunity_H subtype and high immune cell infiltration. ***P < 0.001. [file 12885_2021_9065_MOESM1_ESM.pdf]
